# Pathologies du cuir chevelu chez le Noir africain en dermatologie à Cotonou (Bénin): aspects épidémiologiques et cliniques en fonction du sexe et de l´âge

**DOI:** 10.11604/pamj.2020.37.303.20997

**Published:** 2020-12-02

**Authors:** Bérénice Dégboé, Christiane Koudoukpo, Akimath Habib, Alida Kouassi, Masudi Djodjo, Fabrice Akpadjan, Hugues Adégbidi, Félix Atadokpèdé

**Affiliations:** 1 Service de Dermatologie-Vénérologie, Faculté des Sciences de la Santé, Centre National Hospitalier et Universitaire de Cotonou, Université d´Abomey-Calavi, Cotonou, Bénin,; 2 Service de Dermatologie-Vénérologie, Centre Hospitalier Universitaire Départemental du Borgou-Alibori, Faculté de Médecine, Université de Parakou, Parakou, Bénin,; 3 Centre de Dépistage et de Traitement de la Lèpre et de l´Ulcère de Buruli de Pobè, Fondation Raoul Follereau, Pobè, Bénin

**Keywords:** Pathologies du cuir chevelu, dermatoses alopéciantes, dermatoses non alopéciantes, peau noire, Bénin, Scalp disorders, alopecizing dermatoses, non-alopecizing dermatoses, black skin, Benin

## Abstract

**Introduction:**

les pathologies du cuir chevelu sont fonction de plusieurs facteurs dont l´ethnie, le sexe ou l´âge. Chez le sujet noir, elles peuvent être induites par des facteurs intrinsèques et extrinsèques. Très peu d´études ont été réalisées dans ce groupe ethnique résidant en Afrique noire d´où l´objectif de notre enquête qui était de documenter les aspects épidémiologiques et cliniques des pathologies du cuir cheveu en fonction du sexe et de l´âge dans un service de dermatologie à Cotonou (Bénin).

**Méthodes:**

l´étude était rétrospective et descriptive sur 7 ans et a concerné tous les dossiers des nouveaux patients reçus en consultation dans le service de dermatologie-vénérologie du Centre National Hospitalier et Universitaire (CNHU) de Cotonou dont le motif de consultation était une pathologie exclusive du cuir chevelu. Les caractéristiques épidémiologiques et cliniques ont été saisies et analysées avec le logiciel EPI-Info 7.

**Résultats:**

la prévalence des pathologies du cuir chevelu était de 2,4% (181/7554). Les enfants (0-18 ans) représentaient 38,7%, soit 70 patients et les adultes 61,3%, soit 111 patients. Les enfants de 0-10 ans (54; 29,8%) et les adultes de 25-40 ans (51; 28,2%) étaient les plus touchées. Le sex-ratio était de 1,8. Les dermatoses diagnostiquées étaient non alopéciantes (10; 5,5%) ou alopéciantes (171; 94,5%) dont 82,9% (151/171) de non cicatricielles et 11,7% (20/171) de cicatricielles. Les pathologies les plus fréquentes étaient la teigne (41; 22,6%) prédominant chez les garçons de 0-10 ans, les folliculites chroniques non cicatricielles (39; 21,5%) en majorité chez les garçons de 0-5 ans et les hommes de 19-40 ans, la pelade (38; 21%) dans les deux sexes surtout entre 6-10 ans et 25-40 ans, l´alopécie de traction (17; 9,4%) exclusivement chez les femmes et surtout dans la tranche de 25-40 ans, la folliculite fibrosante de la nuque (12; 6,6%) exclusivement chez les hommes à partir de 19 ans jusqu´à 50 ans, la trichotillomanie (9; 5%) dans les deux sexes, surtout chez les enfants de 6-10 ans et chez les adultes de 25-40 ans, la folliculite épilante de Quinquaud (6; 3,3%) uniformément dans les deux sexes et surtout entre 25-40 ans.

**Conclusion:**

les pathologies du cuir chevelu atteignaient avec prédilection les patients de sexe masculin avant la puberté et les adultes jeunes. Elles étaient réparties par ordre décroissant en dermatoses alopéciantes non cicatricielles, dermatoses alopéciantes cicatricielles et dermatoses non alopéciantes.

## Abstract (en)

**Introduction:**

scalp disorders are related to several factors including ethnicity, gender or age. In black people, they can be caused by intrinsic and extrinsic factors. Very few studies have been conducted in this ethnic group residing in black Africa, hence the purpose of our survey was to highlight the epidemiological and clinical features of age-sex-specific scalp disorders in patients treated in a dermatology department in Cotonou (Benin).

**Methods:**

we conducted a retrospective and descriptive study of all the medical records of new patients coming to consultation in the dermatology department at the National Hospital and University Center (CNHU) of Cotonou over a period of seven years. The main reason for consultation was scalp disorder. Data on epidemiological and clinical features were collected and analyzed with the EPI-Info 7 software.

**Results:**

prevalence of scalp disorders was 2.4% (181/7554). Children (0-18 years) accounted for 38.7% (70 patients) and adults 61.3% (111 patients). Children aged 0-10 (54; 29.8%) and adults aged 25-40 (51; 28,2%) were the most affected. Sex ratio was 1.8. Non-alopecizing dermatosis was diagnosed in 10; 5.5% of cases while alopecizing dermatosis in 171 patients (94.5%), of whom 82.9% (151/171) had non-scarring dermatosis and 11.7% (20/171) had scarring dermatosis. The most common conditions were ringworm (41; 22.6%), mainly occurring in 0-10-year-old boys, chronic non-scarring folliculitis (39; 21.5%) mainly occurring in 0-5-year-old boys and 19-40-year-old men, pelade (38; 21%) occurring in both male and female sexes, especially between the ages of 6-10 and 25-40, traction alopecia (17; 9.4%) occurring exclusively in women and mainly in the 25-40-year-old age group, fibrous folliculitis at the nape of the neck (12; 6.6%) occurring exclusively in men from 19 to 50 years, trichotillomania (9; 5%) occurring in both male and female sexes, mainly in children aged 6-10 years and in adults aged 25-40 years, Quinquaud folliculitis decalvans (6; 3.3%) occurring uniformly in both male and female sexes and mainly between 25-40 years of age.

**Conclusion:**

scalp disorders mainly affect male patients before puberty and young adults. They were arranged in descending order in non-scarring alopecizing dermatoses, scarring alopecia and non-alopecizing dermatoses.

## Introduction

Une chevelure souple sur un cuir chevelu sain, a toujours constitué un symbole de séduction dans toutes les civilisations. Toute pathologie du cuir chevelu peut avoir un impact sur l´image de soi et altérer la qualité de vie [1]. Plusieurs pathologies ont été rapportées dans la littérature comme fréquentes chez les patients ayant une ascendance africaine comparativement aux patients caucasiens. De plus, elles diffèrent selon le sexe et les tranches d´âges [2-4]. Ces pathologies seraient en rapport avec la structure histologique du cheveu Africain [5]. La pathologie du cuir chevelu chez le sujet noir africain est complexe, faisant intervenir des facteurs intrinsèques et extrinsèques à savoir les soins capillaires, les micro-organismes, les affections systémiques ou psychiatriques [6,7]. Très peu d´études ont été réalisées dans ce groupe ethnique résidant en Afrique noire d´où l´objectif de notre enquête qui était de documenter les aspects épidémiologiques et cliniques des pathologies du cuir cheveu en fonction du sexe et de l´âge dans un service de dermatologie à Cotonou (Bénin).

## Méthodes

Nous avons réalisé une étude rétrospective et analytique allant de septembre 2011 à septembre 2017. Elle a concerné tous les dossiers des nouveaux patients reçus en consultation dans le service de dermatologie-vénérologie du Centre National Hospitalier et Universitaire Hubert Koutoukou Maga (CNHU-HKM) de Cotonou dont le motif de consultation était une pathologie du cuir chevelu. Étaient considérées comme pathologies du cuir chevelu, toute dermatose affectant le cuir chevelu et/ou les annexes que sont les glandes et les poils localisés exclusivement sur le cuir chevelu. Nous n´avons pas inclus les autres dermatoses atteignant le revêtement cutané dont l´un des sièges était le cuir chevelu. Le diagnostic de la dermatose du cuir chevelu a été fait essentiellement sur la base de l´anamnèse et de l´examen physique par un dermatologue. Très peu de patients ont réalisé des examens complémentaires tels que l´examen mycologique. Ces pathologies qu´elles soient congénitales ou acquises ont été divisées en deux grands groupes selon qu´elles soient alopéciques ou non. Les dermatoses alopéciques ont été classées selon le mode évolutif cicatriciel ou non. Dans chaque sous-groupe, chaque affection était considérée selon le mécanisme pathogénique: infectieuse, inflammatoire, auto-immun, traumatique ou encore psychologique. Les caractéristiques épidémiologiques et cliniques ont été recueillies à l´aide d´une fiche d´enquête. Les données ont été saisies et analysées avec le logiciel EPI-Info 7.

## Résultats

Au terme de notre enquête, sur 7554 consultants durant la période d´étude, nous avons inclus 181 patients ayant une pathologie localisée exclusivement au cuir chevelu. Ceci correspondant à une prévalence hospitalière de 2,4%. Il y avait 117 patients de sexe masculin contre 64 patients de sexe féminin, donnant une sex-ratio de 1,8. Les enfants (0-18 ans) représentaient 38,7% de l´effectif, soit 70 patients et 77,1% d´entre eux (54 enfants) avaient moins de 10 ans. Les adultes constituaient 61,3% de l´effectif, soit 111 patients et 51 patients (45,9%) avaient entre 25-40 ans. Le Tableau 1 montre la répartition des patients selon l´âge. Les dermatoses alopéciantes étaient retrouvées chez 171 patients soit 94,5% et les dermatoses non alopéciantes chez 10 patients soit 5,5%. Le Tableau 2 résume les principales pathologies diagnostiquées.

**Tableau 1:** répartition selon la tranche d'âge chez les 181 patients atteints de pathologies du cuir chevelu dans le service de dermatologie du CNHU-HKM de Cotonou de septembre 2011 à septembre 2017

	Effectif	Fréquence (%)
0-5	27	14,9
6-10	27	14,9
11-15	10	5,5
16-18	6	3,3
19-24	28	15,5
25-40	51	28,2
41-50	21	11,6
51-60	7	3,9
>60	4	2,2
Total	181	100

**Tableau 2:** répartition des différentes classes d'affections du cuir chevelu chez les 181 patients dans le service de dermatologie du CNHU-HKM de Cotonou de septembre 2011 à septembre 2017

			Effectif	Fréquence (%)
Dermatoses alopéciantes (N=171; 94,5%)	Alopécies non cicatricielles (N=151; 83,4%)	Teigne	41	22,6
		Folliculites chroniques non cicatricielles	39	21,5
		Pelade	38	21,0
		Alopécie de traction	17	9,4
		Trichotillomanie	9	5,0
		Alopécie androgéno-génétique	3	1,7
		Autres*	4	2,2
	Alopécies cicatricielles (N=20; 11%)	Folliculite fibrosante de la nuque	12	6,6
		Folliculite épilante de Quinquaud	6	3,3
		Cellulite disséquante	2	1,1
Dermatoses non alopéciantes (N=10; 5,5%)		Dermite séborrhéique	3	1,7
		Psoriasis	3	1,7
		Lichen	1	0,5
		Eczéma de contact	1	0,5
		Effluvium télogène non alopéciant	1	0,5
		Eczéma séborrhéique	1	0,5
Total			181	100

Autres*: dermite caustique=2; brûlure thermique=2 ; Autres**: eczéma séborrhéique=1

**Figure 1:**
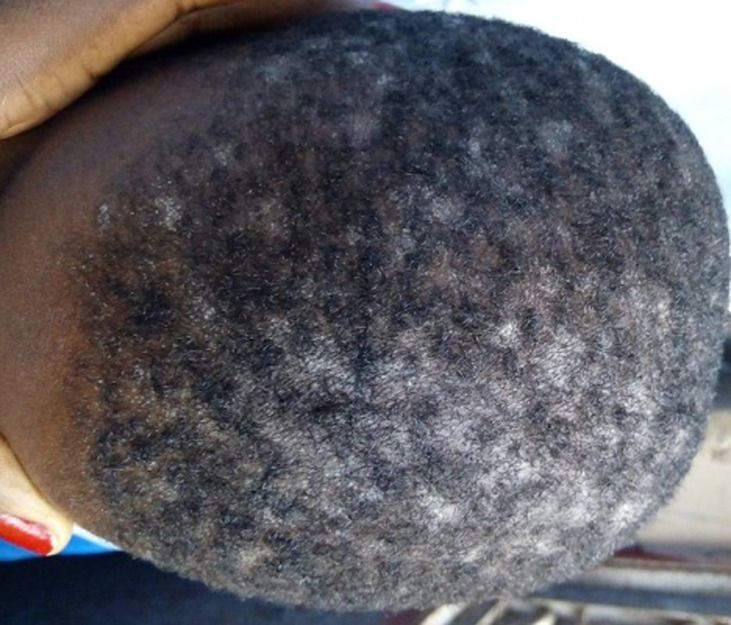
teigne trichophytique du cuir chevelu chez un garçon

**Figure 2:**
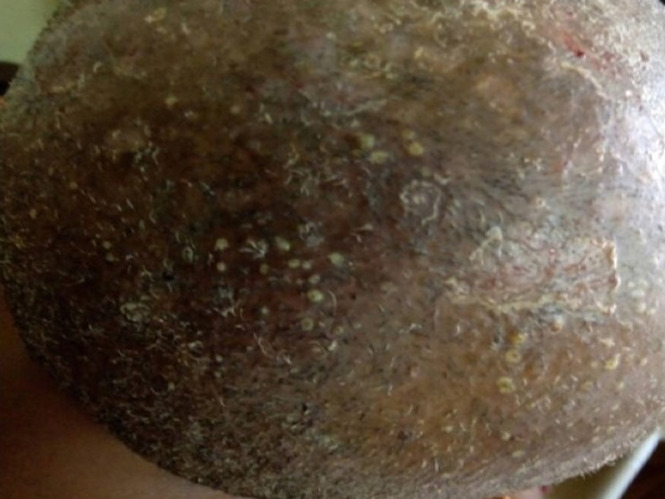
folliculites chroniques du cuir chevelu chez une fille

**Figure 3:**
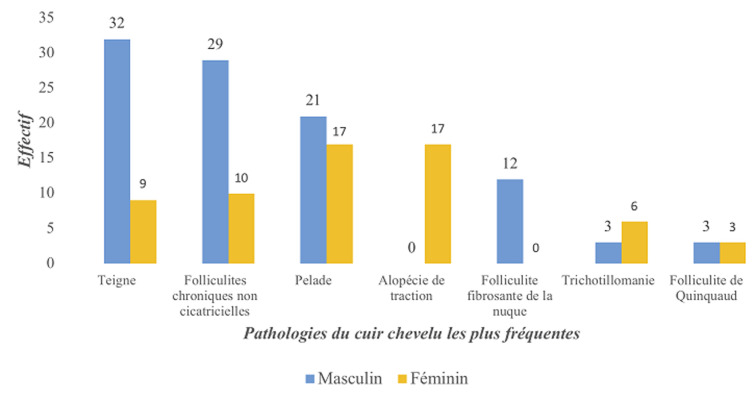
répartition selon le sexe des pathologies du cuir chevelu les plus fréquentes chez les 181 patients dans le service de dermatologie du CNHU-HKM Cotonou de septembre 2011 à septembre 2017

**Figure 4:**
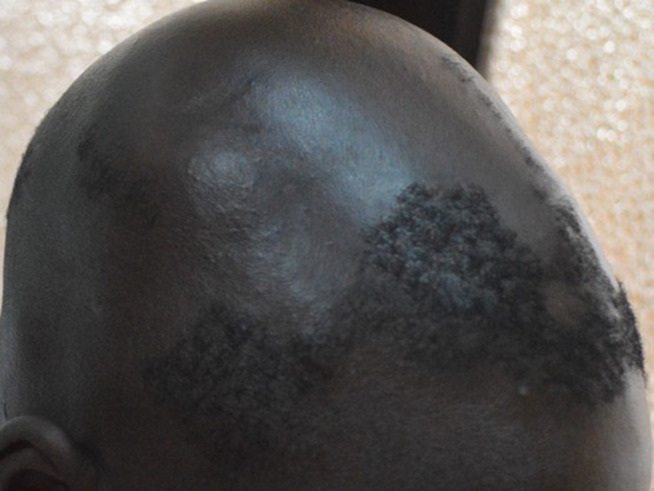
pelade ophiasique du cuir chevelu chez un garçon

**Figure 5:**
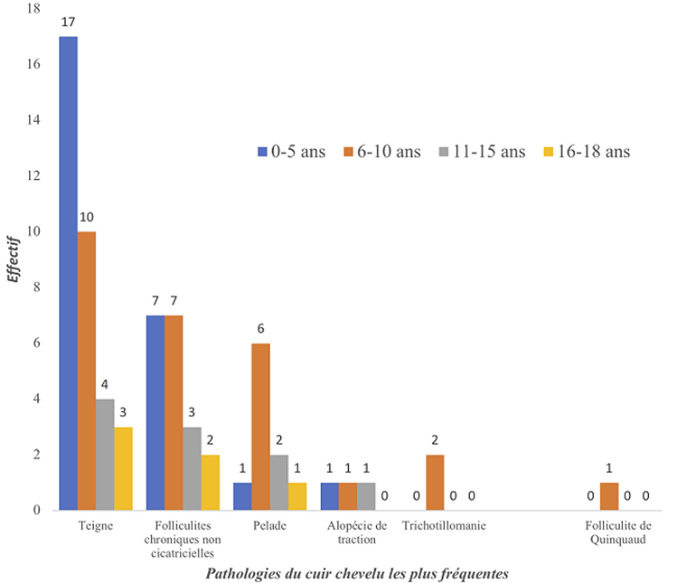
répartition des pathologies du cuir chevelu les plus fréquentes chez les 70 enfants dans le service de dermatologie du CNHU-HKM Cotonou de septembre 2011 à septembre 2017

**Figure 6:**
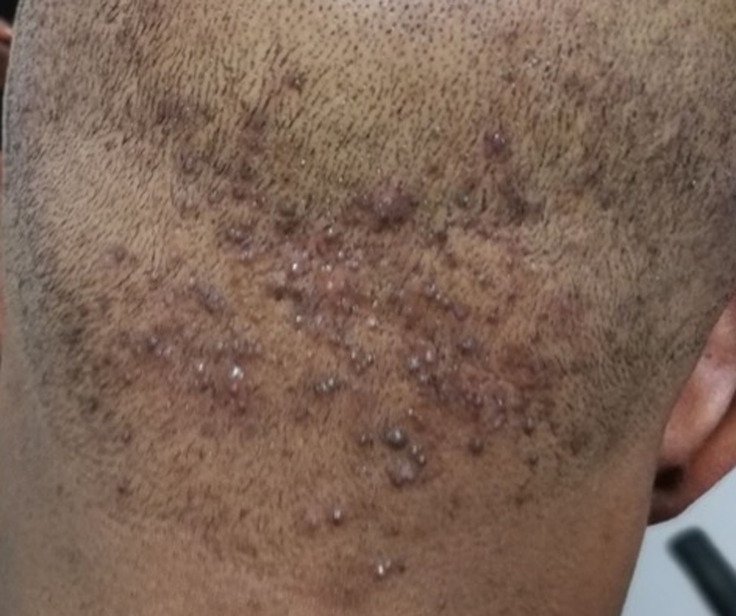
folliculite fibrosante de la nuque chez un homme

**Figure 7:**
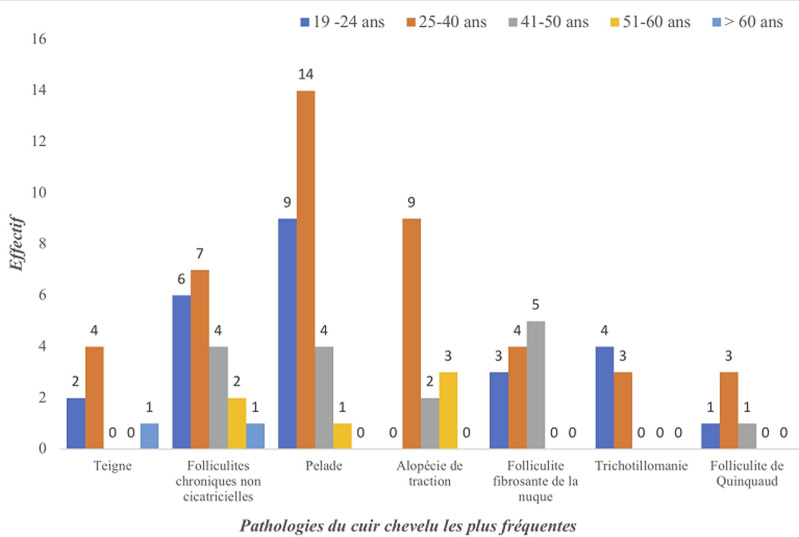
répartition des pathologies du cuir chevelu les plus fréquentes chez les 111 adultes dans le service de dermatologie du CNHU-HKM de Cotonou de septembre 2011 à septembre 2017

La teigne (Figure 1) et les folliculites chroniques non cicatricielles (FCNC); illustrées par la Figure 2, étaient plus fréquentes chez les patients de sexe masculin. La Figure 3 montre la répartition des diverses pathologies en fonction du sexe. Chez les enfants, les pathologies les plus fréquentes étaient la teigne (34/70; 48,6%), les FCNC; (19/70; 27,1%) et la pelade (10/70; 14,3%) (Figure 4). Les enfants entre 0-10 ans étaient les plus touchés quelle que soit la pathologie (Figure 5). Au sein des adultes, les causes les plus fréquentes de chute de cheveux étaient la pelade avec 28 cas (25,2%), les FCNC avec 20 patients (18%), l´alopécie de traction avec 14 cas (12,6%). Pour ces trois premières affections, les patients entre 25-40 ans étaient les plus touchés. La folliculite fibrosante de la nuque (FFN); illustrée par la Figure 6, était l´apanage des adultes. Elle a été retrouvée chez 12 patients, soit 10,8% des adultes. La répartition de ces différentes affections est décrite sur la Figure 7.

La teigne était significativement fréquente chez les garçons entre 0-5 ans (p=0,04). Les FCNC étaient retrouvées tant chez les enfants (19 cas) que chez les adultes (20 cas). La pelade était observée quel que soit le sexe mais plus chez les adultes entre 25-40 ans (p=0,98). L´alopécie de traction (AT) était exclusivement retrouvée chez les femmes, particulièrement entre 25-40 ans (p=0,90). La FFN était l´apanage des hommes entre 19 ans et 50 ans.

## Discussion

Les pathologies du cuir chevelu chez les sujets noirs étaient peu fréquentes dans le service de dermatologie à Cotonou. Cette prévalence pourrait donc être nettement inférieure à celle dans la population générale. Ceci pourrait être expliquée d´une part, par le manque d´accessibilité ou la banalisation de certaines affections du cuir chevelu. D´autre part, certains patients ignorent que les dermatologues soignent les pathologies du cuir chevelu et consultent plutôt les coiffeurs, les coiffeuses et les pharmaciens. A ce jour, divers travaux menés en Afrique noire: au Sénégal [8] au Nigéria [7] en Afrique du Sud [9] au Burkina Faso [10] et en France [11] retrouvent ce faible taux de consultants pour les affections du cuir chevelu. Une étude prospective, en population générale serait indiquée pour mieux apprécier la prévalence des pathologies du cuir chevelu chez le noir africain.

Il y avait une nette prédominance masculine. Les deux sexes sont affectés par les pathologies du cuir chevelu mais la sex-ratio varie en fonction du type d´étude [4,8,12]. Comme dans la plupart des études réalisées en Afrique noire, la teigne était la principale étiologie des dermatoses infectieuses [13-15]. Elle est plus fréquente entre 0-10 ans [16]. Nos résultats confirment que chez les enfants, les affections du cuir chevelu les plus fréquentes sont: la teigne, la pelade, l´alopécie de traction et la trichotillomanie [15,17]. Les folliculites chroniques du cuir chevelu constituent une entité nosologique très peu étudiée. Leur classification est confuse, parfois controversée [18]. Elles comprennent les formes cicatricielles regroupant entre autres la folliculite décalvante ou épilante de Quinquaud, la cellulite disséquante, la folliculite en touffe, la FFN [19], et les formes non cicatricielles qui sont encore mal individualisées [20].

Les FCNC ont été retrouvées dans notre série chez les enfants préférentiellement entre 0 et 10 ans et chez les adultes surtout entre 19 et 40 ans. Ce groupe de folliculites constitue une entité encore floue et complexe. Plusieurs étiologies ont été rapportées; infectieuses [21-23] ou non infectieuses [20,24]; elles sont observées chez l´enfant comme chez l´adulte. Dans notre contexte, une origine bactérienne est souvent suspectée. Cette hypothèse est soutenue par l´absence d´une décontamination efficace des accessoires de coiffure le plus souvent utilisés en commun. La pelade est une maladie auto-immune survenant des fois sur un terrain génétiquement prédisposé [25]. Les facteurs environnementaux tels que le stress, les fluctuations hormonales, les agents infectieux, la vaccination ou le régime alimentaire jouent probablement un rôle dans le déclenchement, la sévérité ou l´évolution de l´affection [26,27]. Elle survient autant chez les enfants que chez les adultes et indépendamment du sexe. Dans notre série, elle était plus fréquente chez les adultes. La trichotillomanie est la traduction d´un trouble psychologique à l´origine de l´arrachement du poil [26,28]. Elle a été rapportée chez les jeunes adultes entre 19 ans et 40 ans, démontrant ainsi, comme dans la pelade, les intrications entre le système psychique et le cuir chevelu. La pelade et la trichotillomanie étaient fréquentes chez les enfants en âge scolaire. Ceci pourrait être en rapport avec le stress généré par la vie scolaire ou encore la peur de perdre un parent dans cette tranche d´âge [26,27,29].

Certaines pathologies touchent préférentielle ment, voire exclusivement les Africains ou les populations d´ascendance africaine. Elles sont favorisées par les caractéristiques intrinsèques liées à la structure, la croissance et la densité du cheveu crépu. En effet, on distingue classiquement 3 grands groupes de cheveux: le cheveu asiatique, le cheveu européen et le cheveu africain [30]. Le cheveu africain est crépu, noir, plus courts et moins denses. Les follicules pilaires ont une implantation dermique profonde presque horizontale et les tiges pilaires qui en sont issues ont une section elliptique ou aplatie et un trajet en hélice serrée dont la spirale s´amorce avec l´émergence du cheveu à la surface cutanée. L´ultrastructure du cheveu crépu spiralé majore le risque de nœuds et de fracture du cheveu lors du peignage et des tresses [31]. Les principales causes sont les agressions physiques et chimiques que doivent subir cette chevelure pour être mise en forme selon les canons esthétiques de la mode et les critères d´acceptation sociale de la coiffure [1-6,32].

L´AT est l´une de ces pathologies, particulièrement fréquente chez les femmes [8-10]. Elle a été observée effectivement chez les patients de sexe féminin dans notre étude, tôt chez les enfants de moins de 15 ans, mais surtout chez les adultes entre 25 et 40 ans. Kluger *et al*. estiment que les enfants présentent la forme aiguë alors que les adultes développent la forme chronique [33]. Un diagnostic et un traitement précoces s´avèrent importants en raison des implications esthétiques et sociales et du cercle vicieux qu´elle entraîne. En effet les patientes utilisent les extensions et foulards pour camoufler l´alopécie, mais ces gestes ne font qu´aggraver la pathologie [5,32,33].

A l´opposé de l´AT, la FFN encore appelée acné chéloïdienne de la nuque est une pathologie qui survient préférentiellement chez les hommes. Elle est aussi liée à la constitution et le mode de croissance du cheveu crépu. Elle survient rarement dans l´enfance et au-delà de 50 ans [34,35]. Ce fait a été confirmé par notre étude où la FFN était retrouvée exclusivement chez les hommes entre 19 ans et 50 ans. Ces observations ont emmené à suggérer comme facteurs favorisants, à part le rasage du cuir chevelu, l´hyperandrogénie, l´hyperséborrhée, l´inflammation chronique du follicule pilo-sébacé [34-36]. Une étude récente a montré que d´autres pathologies du follicule pilo-sébacé telles que la dermite séborrhéique, l´acné, la pseudo-folliculite de la barbe, la folliculite chronique du cuir chevelu ou la folliculite décalvante peuvent être associées. De même qu´un syndrome métabolique incluant un diabète, une hypertension artérielle ou une obésité a été retrouvé associé [37].

## Conclusion

Les pathologies du cuir chevelu chez le Noir africain dans le service de dermatologie du CNHU-HKM de Cotonou étaient multivariées, atteignant tous les âges et surtout le sexe masculin. Elles étaient réparties par ordre décroissant en dermatoses alopéciantes non cicatricielles, dermatoses alopéciantes cicatricielles et dermatoses non alopéciantes. La teigne, les folliculites chroniques non cicatricielles et la pelade étaient les étiologies les plus fréquentes chez les enfants. Chez les adultes, la pelade, les folliculites chroniques non cicatricielles, l´alopécie par traction et la folliculite fibrosante de la nuque étaient les principales pathologies observées.

### Etat des connaissances sur le sujet

- Les pathologies du cuir chevelu sont fréquentes chez les sujets à carnation foncée;
- Le mécanisme pathogénique met en cause des facteurs intrinsèques liés à la structure histologique du poil et des facteurs extrinsèques;
- La teigne représente la pathologie du cuir chevelu la plus fréquente chez les enfants en Afrique noire.

### Contribution de notre étude à la connaissance

- Les patients consultent rarement en dermatologie pour les pathologies du cuir chevelu;
- Les sujets de sexe masculin consultent plus souvent pour des pathologies du cuir chevelu;
- Ces pathologies varient tant chez les hommes que chez les femmes, mais aussi chez l´enfant et l´adulte.
